# Accidental Overdose of Paliperidone Palmitate

**DOI:** 10.1155/2019/7406298

**Published:** 2019-04-11

**Authors:** Chiedozie Ojimba, Ayotomide Oyelakin, Taher Khandaker

**Affiliations:** Department of Psychiatry, Interfaith Medical Center, Brooklyn, NY, USA

## Abstract

Long-acting injectable (LAI) antipsychotics first introduced in 1960s are useful in the treatment of schizophrenic patients with poor medication adherence due to their maintaining feature of therapeutic plasma level without daily administration. Paliperidone Palmitate is one of such LAI antipsychotic drugs used due to its benefit of maintaining a therapeutic plasma level with four-week interval of injections. We report the case of a 21-year-old male with a history of mental illness that presented with selective mutism, disorganized speech, thought process and behavior, and auditory hallucinations who accidentally received 624 mg Paliperidone Palmitate intramuscularly with no reported side effects after 2 weeks of monitoring and observation. Paliperidone is a D2, 5HT2A receptor antagonist with additional antagonist activity at *α*-1 and *α*-2, H-1 receptor sites, and four metabolic pathways identified for its metabolism. Studies have reported adverse effects such as acute dystonia, acute renal failure, and cardiovascular abnormalities with Paliperidone overdose; however there is no reported literature on Paliperidone Palmitate overdose, though there have been reported cases of Paliperidone Palmitate side effects of hypersexuality and angioedema with the standard dose.

## 1. Introduction

Long-acting injectable (LAI) antipsychotics are useful in the treatment of schizophrenic patients with poor medication adherence due to their maintaining feature of therapeutic plasma level without daily administration. However, their long-lasting property can cause complicated long-lasting side effects [1]. Paliperidone Palmitate is one of the long-acting injectable (LAI) antipsychotic drugs used for schizophrenic patients with poor medication adherence. It has the benefit of maintaining a therapeutic plasma level with a four-week interval of injections [1]. Long-acting injectable antipsychotics help to achieve better adherence by replacing the need for taking daily oral antipsychotic medication. In addition, there is no first-pass hepatic metabolism, which contributes toward a more predictable bioavailability [2]. Paliperidone or 9-hydroxyrisperidone is the major active metabolite of Risperidone. It was approved by FDA in December 2006 for the acute and maintenance treatment of schizophrenia and schizoaffective disorder and may be used as monotherapy or as an adjunct to mood stabilizers and/or antidepressants [3]. In a six-week efficacy trial prior to FDA approval, the adverse reactions that were reported more commonly than with placebo included tachycardia (9%–22%), hyperkinesias (3%–11%), hypertonia (1%–6%), extrapyramidal symptoms (EPS) (3%–10%), and somnolence (4%–13%) [2]. To date, there are no literature reports on Paliperidone overdose and its management to avoid reported side effects or adverse drug reactions. We report a case of accidental intake of 624 mg intramuscular injection of Paliperidone Palmitate with no reported side effects after 2 weeks of monitoring and observation.

## 2. Case Summary

Patient is a 21-year-old African American male, single, unemployed, and homeless with a reported history of mental illness and no past medical history who was brought in by ambulance from the shelter for psychiatry evaluation as the patient was reportedly found talking to himself. Upon evaluation in the psychiatry emergency room (ER), he was uncooperative and selectively mute. He reported recent relocation to New York from California 2-3 months prior to presentation; however, the exact duration of his symptoms could be not elicited and no valid collateral could be obtained. He reported he was on Olanzapine 10 mg orally daily and needed to get a refill. Patient was grossly disorganized in his speech, thought process, and behavior. He was internally preoccupied; he was seen smiling and mumbling to self. His mentation was slowed and he had difficulty processing information with poor reality testing. Urine toxicology was negative for any illicit substance on admission. He was admitted to the inpatient psychiatry unit for stabilization after receiving a stat dose of Olanzapine 5 mg orally in the psychiatry emergency room. On the inpatient unit, patient was started on the Olanzapine 20 mg orally at bedtime for psychosis, Valproic acid 500 mg orally twice daily for mood stabilization due to agitation and low dose, regular form of Trazodone 100 mg orally at bedtime for sleep on day 1 of his hospital course. Patient was compliant with his medication and after 2 weeks of compliance with Olanzapine, he remained grossly disorganized and internally preoccupied, constantly seen responding to internal stimuli. His medications were reviewed, Olanzapine cross-tapered with Risperidone on day 14 with the aim of giving him Paliperidone Palmitate (Invega Sustenna) if the patient improves on a trial of Risperidone. He was started on Risperidone 1 mg orally twice daily which he tolerated with no report of adverse effect. On day 17, he was given the first dose of Invega Sustenna 234 mg intramuscularly (IM), well tolerated with no report of side effect or adverse drug reaction. He received the second dose of Invega Sustenna 156 mg IM five days after the first dose on day 22, also well tolerated with no report of side effect or adverse drug reaction. On day 28, six days after he received the second dose of Invega Sustenna, patient received another dose of Invega Sustenna 234 mg IM due to name error. Patient was mistaken for another patient who bears the same last name. Patient was immediately evaluated, given Benztropine 2 mg IM stat for extrapyramidal symptom prevention.

The patient was closely observed for extrapyramidal and dose-related side or adverse effects such cardiac arrhythmias, QTc prolongation, dizziness, extrapyramidal symptoms, and hypotension. Patient had stat electrocardiogram (EKG), complete blood count, and complete metabolic profile which were normal. Carbamazepine 400 mg orally stat then 400 mg every 8 hours was commenced the following day with the aim of decreasing serum levels of Paliperidone. Serial daily EKG was ordered for monitoring of QTc, and vital signs were checked every 15 minutes. Patient was also instructed to report any unusual or abnormal feelings which could be related to the high dose of Paliperidone Palmitate or side effects associated with hyperprolactinemia. Serum prolactin level was not done as patient had no report of side effects related to hyperprolactinemia. After 2 weeks of close monitoring, patient did not report any of the reported side effects of Paliperidone Palmitate and none was observed by the treatment team. Patient was discharged on day 55 of admission with no reports or observed side effects associated with Paliperidone Palmitate.

## 3. Discussion 

Conventional antipsychotic depot formulations were first introduced in the 1960s as a means to improve compliance with therapy. However, their high propensity to cause extrapyramidal symptoms and raise prolactin levels has limited their use over time. In recent years, research and development by the pharmaceutical companies have succeeded in formulating new antipsychotic medications known as atypical antipsychotics. Paliperidone Palmitate is one of the most recent atypical antipsychotics to be developed as a long-acting injection and was approved by the US Food and Drug Administration in August 2009 for acute and maintenance therapy in adult patients with schizophrenia [3, 4]. Paliperidone, also described as 9-hydroxyrisperidone, is the major active metabolite of Risperidone [3, 4]. Its pharmacology and mechanism of action are therefore believed to be similar to those of Risperidone. Paliperidone acts as an antagonist at dopamine D2 and serotonin 5HT2A receptors, exhibiting a high 5HT2A: D2 affinity ratio, similar to other atypical agents. It also has binding activity as an antagonist at *α*1- and *α*2-adrenergic receptor sites and H1-histaminergic receptors but virtually has no affinity for cholinergic receptors. Paliperidone activity profile suggests that it has the potential to cause orthostatic hypotension, weight gain, and sedation. However, because it has no antagonistic activity at cholinergic receptors, it has a low propensity to cause anticholinergic adverse effects and cognitive impairment [3, 4]. Paliperidone is largely excreted unchanged in the urine. Four metabolic pathways have been identified for the metabolism of Paliperidone including dealkylation, hydroxylation, dehydrogenation, and benzisoxazole scission, although none accounted for more than 10% of the oral dose administered [4]. While cytochromes P450 (CYP) 2D6 and CYP3A4 have been implicated in the metabolism of Paliperidone in* in vitro* studies, these isoenzymes play a limited role in the metabolism of Paliperidone* in vivo* [4]. Paliperidone is not metabolized by CYP 2D6 cytochrome system [3]. The nanocrystal molecules which make up the Paliperidone Palmitate suspension also allow it to undergo slow dissolution, yielding a half-life of 25–49 days [1, 4]. Treatment-emergent adverse effects (TEAEs) occurring more frequently in the Paliperidone Palmitate group than in the placebo group included insomnia, headache, dizziness, sedation, vomiting, injection site pain, extremity pain, myalgia, and extrapyramidal symptoms [4].

There have been case reports of Paliperidone overdose with their associated adverse effects such as acute dystonia, acute renal failure, and cardiovascular side effects such as tachycardia, hypotension, and dizziness [4–6]. To date, there is no reported literature on Paliperidone Palmitate overdose; rather there have been reported cases of Paliperidone Palmitate side effects of hypersexuality and angioedema from normal required dose but not from overdose [5, 7]. Our case is that of a patient who took a total dose of 624 mg of Paliperidone Palmitate within a space of 11 days in a staggered manner. The patient did not report any of the postmarketing dose-related adverse reactions or side effects. Serial monitoring of vital signs did not reveal any tachycardia or hypotension. Daily EKG monitoring for 10 days showed the lowest and highest QTc as 393 and 434, respectively. QTc of 393 was on the 11th day after he received the third dose of Paliperidone Palmitate, 234 mg IM, while 434 was on the first day after the erroneous injection. The baseline EKG was 413 on the day of admission. The patient did not report any extrapyramidal side effects and none were observed by the treatment team. It is unclear why our patient did not exhibit any side or adverse effects; however, it could be that the Carbamazepine he received played a role, although studies have shown that there is little or no* in vivo* effects of CYP 2D6 on Paliperidone. It could be that our patient is a fast metabolizer of Paliperidone through one of the four different methods listed previously; unfortunately, no genomic polymorphism test was done to confirm this.

## 4. Conclusion

Paliperidone dose-related side or adverse effects can be fatal; hence it is imperative to take extra precaution to avoid accidental overdose. Close monitoring of the patient is important as there have been reported delayed side effects.
